# Quantitative assessment of neurodevelopmental maturation: a comprehensive systematic literature review of artificial intelligence-based brain age prediction in pediatric populations

**DOI:** 10.3389/fninf.2024.1496143

**Published:** 2024-11-12

**Authors:** Eric Dragendorf, Eva Bültmann, Dominik Wolff

**Affiliations:** ^1^Peter L. Reichertz Institute for Medical Informatics of TU Braunschweig, Hannover Medical School, Hannover, Germany; ^2^Institute of Diagnostic and Interventional Neuroradiology, Hannover Medical School, Hannover, Germany

**Keywords:** pediatric, children, artificial intelligence, brain age gap, brain age prediction, magnetic resonance imaging, computed tomography, electroencephalography

## Abstract

**Introduction:**

Over the past few decades, numerous researchers have explored the application of machine learning for assessing children’s neurological development. Developmental changes in the brain could be utilized to gauge the alignment of its maturation status with the child’s chronological age. AI is trained to analyze changes in different modalities and estimate the brain age of subjects. Disparities between the predicted and chronological age can be viewed as a biomarker for a pathological condition. This literature review aims to illuminate research studies that have employed AI to predict children’s brain age.

**Methods:**

The inclusion criteria for this study were predicting brain age via AI in healthy children up to 12 years. The search term was centered around the keywords “pediatric,” “artificial intelligence,” and “brain age” and was utilized in PubMed and IEEEXplore. The selected literature was then examined for information on data acquisition methods, the age range of the study population, pre-processing, methods and AI techniques utilized, the quality of the respective techniques, model explanation, and clinical applications.

**Results:**

Fifty one publications from 2012 to 2024 were included in the analysis. The primary modality of data acquisition was MRI, followed by EEG. Structural and functional MRI-based studies commonly used publicly available datasets, while EEG-based studies typically relied on self-recruitment. Many studies utilized pre-processing pipelines provided by toolkit suites, particularly in MRI-based research. The most frequently used model type was kernel-based learning algorithms, followed by convolutional neural networks. Overall, prediction accuracy may improve when multiple acquisition modalities are used, but comparing studies is challenging. In EEG, the prediction error decreases as the number of electrodes increases. Approximately one-third of the studies used explainable artificial intelligence methods to explain the model and chosen parameters. However, there is a significant clinical translation gap as no study has tested their model in a clinical routine setting.

**Discussion:**

Further research should test on external datasets and include low-quality routine images for MRI. T2-weighted MRI was underrepresented. Furthermore, different kernel types should be compared on the same dataset. Implementing modern model architectures, such as convolutional neural networks, should be the next step in EEG-based research studies.

## 1 Introduction

Artificial intelligence has the potential to support clinical decision-making. This has been demonstrated in a study that revealed similar diagnostic performance between artificial intelligence and clinicians in detecting fractures (Kuo et al., 2022). However, compared to fracture detection, brain development estimation is more complex. In adults, aging was found to impact the cerebral composition as grey matter decreases and white matter increases (Allen et al., 2005). For children, the brain matures in an orchestrated and predetermined matter, continues after birth, and correlates with infants’ skills. Anatomical changes such as increased myelination, changes in volume, and cortex thickness can be tracked by multiple modalities. For this, ultrasound, computed tomography (CT), and magnetic resonance imaging (MRI) are viable options, with MRI being the most versatile as it reflects folding and the myelination status (Allen et al., 2005; Stiles and Jernigan, 2010). Brain development is an intricate process caused by structural and functional changes in children (Stiles and Jernigan, 2010; Lenroot and Giedd, 2006).

Eight weeks post-conception, the human brain is mainly developed, with the central and the peripheral nervous system being defined. However, the brain needs further refinement. The rudimental pathways that developed become more robust, and the rapid growth of cortical and subcortical structures continues (Stiles and Jernigan, 2010). In addition, the complexity of the brain changes by neuronal migration and synaptogenesis. The last trimester of pregnancy and the first two postnatal years are characterized by an exponential increase in surface area due to the development of gyri and sulci and a tremendous increase in grey and white matter (Barkovich, 2005). After birth, myelination increases significantly until the first two years, thus increasing neuronal transmission speed. The change in myelination of specific brain regions can be charted in milestones reached at a certain age. Generally, the infant’s brain maturation status adapts to the adult pattern (Barkovich, 2005; Staudt et al., 2000). The changes caused by a decreasing brain water content and an increase in myelination are indirectly reflected by MRI and can be perceived using T1- and T2-weighted MRI (Abdelhalim and Alberico, 2009). In the context of infant MRI scans, T1-weighted images provide valuable insights into maturation status during the first 6 to 8 months by focusing on T1 signal increase, whereas T2-weighted images are most effective for assessing infants between 6 and 18 months of age (Barkovich, 2005; Abdelhalim and Alberico, 2009). On T2-weighted images, signal reduction of the structures occurs with increasing myelination. There, adult appearance is reached at approximately 18 months of age (Barkovich, 2005; Staudt et al., 2000). Further, the total brain volume increases as white and grey matter growth continues until reaching the age of 6 years. After that, only white matter increases, while grey matter decreases (Dubois et al., 2021). In a clinical setting, children suspected of having neurodevelopmental or intellectual disabilities receive special attention and undergo further investigation. In addition to checking the developmental, birth, social, and family history, a physical examination will be pursued. Children with an abnormal examination status eventually receive neuroimaging (Etzion et al., 2017).

This highlights the complexity of predicting age in children and presents an opportunity for early medical interventions. Identifying cerebral changes related to maturation to estimate the age with high accuracy and precision could become automated, which is beneficial for clinical routine. This automatization could identify neurodevelopmental disorders, track progress, and start interventions early in a clinical setting. Currently, estimating brain age necessitates the expertise of specifically trained personnel. Automated brain age prediction has the potential to serve as a cost-effective diagnostic tool that offers support to clinical personnel. This could be approached by determining an individual’s brain development based on artificial intelligence (AI). It could analyze data from MRI scans or measurements from an electroencephalogram (EEG), as they indirectly reflect the cytological and neurostructural changes (Barkovich, 2005; Dubois et al., 2021; Tierney and Nelson, 2009; Scher, 2017). The predicted age by the model for a certain individual is called brain age and is considered a relevant neurodevelopmental biomarker compared to the individual’s chronological age (Franke and Gaser, 2019). For several years, many researchers have tried to predict the brain age via artificial intelligence and used different approaches of either healthy or diseased individuals (Franke and Gaser, 2019). Studies on a healthy population pose a crucial step in determining the accuracy and precision of the predictive algorithm. Once the age can be predicted for healthy participants, the predicting algorithm can be used on diseased patients to estimate the so-called “brain age gap” or “brain age delta.” This is performed by subtracting the predicted brain age from the chronological age. The assumption states that a negative brain age means delayed brain development or neurodegeneration and vice versa (Franke and Gaser, 2019). Diseases that decelerate the brain’s physiological development could be identified and treated early, thus improving the outcome. However, data collection in children is more challenging because sedation is usually needed to decrease movement artifacts. Especially newborns and young children tend to move and are often sedated for brain imaging to reduce movement artifacts that need to be removed via postprocessing (Barkovich, 2005; Abdelhalim and Alberico, 2009; Dubois et al., 2021).

Authors experiment with different modalities and model types to find the best combination for age prediction. To our knowledge, there is no review covering the topics discussed in this review. So, this literature review aims to present an overview of methods used for brain age determination by artificial intelligence in a healthy pediatric population. It highlights age groups’ different data acquisition approaches and compares the quality of the respective methods. We ought to find patterns and discuss the relevance of deviations to ease the way for other researchers into this highly important topic.

## 2 Methods

The Literature in PubMed and IEEEXplore was searched to present the spectrum of approaches taken for predicting brain age in children with the help of artificial intelligence following the PRISMA guidelines. Publications that describe AI models used to predict brain age in a healthy population of children from birth up to 12 years were included. Literature with participants older than 12 years was only included if the dataset partially included younger participants. Studies meeting the inclusion criteria and building an algorithm on a healthy population but testing on a diseased population were included. Review articles were excluded. The results of the model predicting the healthy cohort were reported in the included articles. In addition, unpublished articles, such as preprints, were excluded to ensure quality. The search was performed on April 2nd, 2024, and the chosen language for literature was English. The two databases, PubMed and IEEEXplore, allow the addition of logical expressions into the search term and bundle synonyms in parenthesis. These databases were used to search for literature containing the words “paediatric,” “artificial intelligence,” and “brain age” and synonyms for each. All synonyms for the respective umbrella terms can be found in Table 1. The search terms differed for each database as the search algorithm needed specific adjustments. PubMed allows the inclusion of mesh words and restrictions to the search for titles and abstracts only. To do so, the following search term was created for PubMed:

**Table 1:** Synonyms used for search terms.

**paediatric**	**artificial intelligence**	**brain age**
child*	neural net*	brain growth
infant	machine learning	brain development
pediatric	deep learning	brain maturation
	AI	brain-age-gap
		brain age gap
		predicted age difference

The first row in bold shows the umbrella terms used for finding synonyms. The asterisk represents any group of characters, including no character, and appends the root of a word (e.g., child* includes children and child).

((“child*”[Title/Abstract]) OR (“infant”[Title/Abstract]) OR (“pediatric*“[Title/Abstract]) OR (“paediatric*“[Title/Abstract])) AND ((“Artificial Intelligence”[Mesh]) OR (“neural net*”[Title/Abstract]) OR (“artificial intelligence”[Title/Abstract]) OR (“machine learning”[Title/Abstract]) OR (“Deep Learning”[Title/Abstract]) OR (“AI”[Title/Abstract])) AND ((“Brain growth”[Title/Abstract]) OR (“Brain development”[Title/Abstract]) OR (“brain age”[Title/Abstract]) OR (“brain maturation”[Title/Abstract]) OR (“brain-age-gap”[Title/Abstract]) OR (“brain age gap”[Title/Abstract]) OR (“brain maturity”[Title/Abstract]) OR (“predicted age difference”[Title/Abstract]) OR (“Brain/growth and development”[Mesh]))

In addition, filters for human species and age range up to 12 years were set. Thus, for age, the following options were selected: “Child: birth-18 years,” “New-born: birth-1 month,” “Infant: birth-23 months,” “Infant: 1–23 months,” “Preschool Child: 2–5 years,” “Child: 6–12 years.”

IEEEXplore does not contain MESH categorization and needs other adjustments to restrict the search algorithm to title and abstract, resulting in the following search term:

(“Abstract”:child* OR “Abstract”:infant OR “Abstract”:pediatric* OR “Abstract”:paediatric* OR “Document Title”:child* OR “Document Title”:infant OR “Document Title”:pediatric* OR “Document Title”:paediatric*) AND (“Abstract”:"Artificial Intelligence” OR “Abstract”:"neural net*” OR “Abstract”:"machine learning” OR “Abstract”:"Deep Learning” OR “Abstract”:AI OR “Document Title”:“Artificial Intelligence” OR “Document Title”:"neural net*” OR “Document Title”:"machine learning” OR “Document Title”:"Deep Learning” OR “Document Title”:AI) AND (“Abstract”:"Brain growth” OR “Abstract”:"Brain development” OR “Abstract”:"brain age” OR “Abstract”:"brain maturation” OR “Abstract”:"brain-age-gap” OR “Abstract”:"brain age gap” OR “Abstract”:"brain maturity” OR “Abstract”:"predicted age difference” OR “Document Title”:"Brain growth” OR “Document Title”:"Brain development” OR “Document Title”:"brain age” OR “Document Title”:"brain maturation” OR “Document Title”:"brain-age-gap” OR “Document Title”:"brain age gap” OR “Document Title”:"brain maturity” OR “Document Title”:"predicted age difference”)

There were no additional filters available in IEEEXplore.

**Figure 1:**
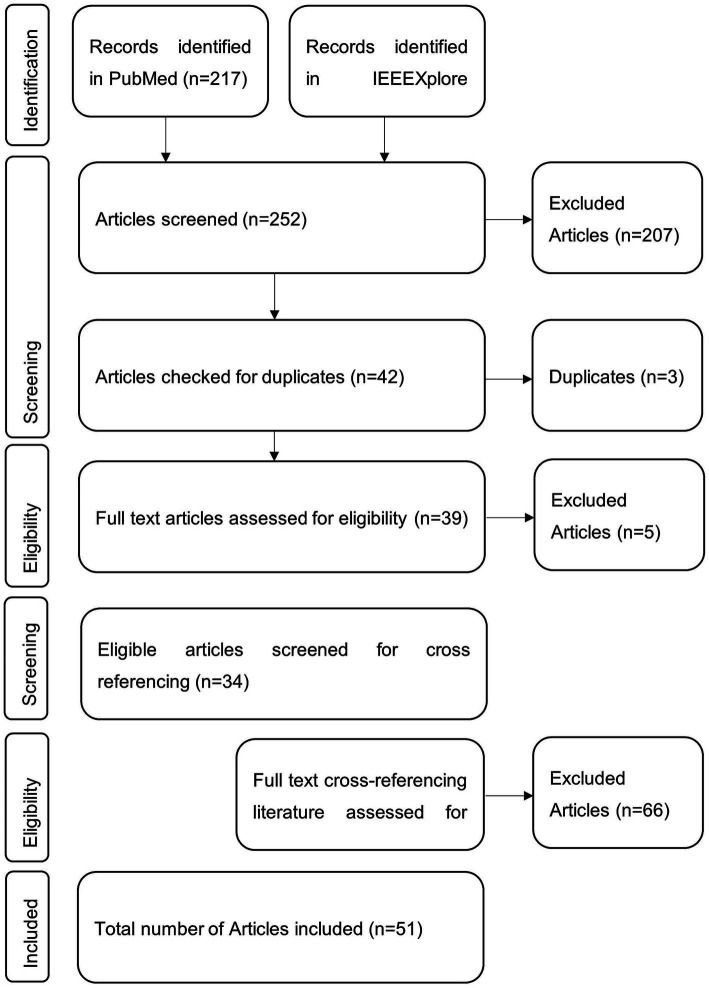
Visual representation of the publication selection procedure. Literature was searched via the databases PubMed and IEEEXplore with the search query. Eligible and thus included items were further searched for fitting cross-references.

All database results were downloaded, manually checked for eligibility, and included if inclusion criteria were met. Afterward, the literature was checked for references suitable for inclusion in this review. These were again checked for eligibility. The references mentioned in the cross-referencing literature were not investigated any further. The selection procedure is visualized in Figure 1.

In analyzing literature, a particular focus was set on the following aspects. The methods section was scanned by one author for data acquisition, age range, sex, preprocessing, and AI model type. Studies that referred to online resources were followed via the given reference or searched via Google. If authors referred to Supplementary materials, those were included. All results were transferred manually to Excel. Data acquisition was examined, as it is essential to know what technology was used to obtain data and thus build the model’s foundation. In the next step, age ranges were manually extracted from the literature. We then analyzed whether specific age ranges were not covered. Then, the age ranges were processed using Python (kernel 3.12.4) and the libraries Matplotlib (version 3.9.2), NumPy (version 1.26.4), and Pandas (version 2.2.2) for visualization. To identify similarities and to establish a typical workflow, the data pre-processing steps were manually transferred to Excel and clustered for each modality, CT, MRI, rs fMRI, and EEG, to inspect patterns. The frequency of different artificial intelligence types and their respective quality for age prediction were also clustered and processed for visual inspection in Excel. In articles that used more than one model type, the best performing was chosen to present the quality in this article. Whenever it was compared to a specific type or the aim of the study had a comparison in mind, all models were shortly discussed. All models were included in the graphical representation and the frequency. For the quality assessment, we included varying measurements, such as mean absolute error and correlation coefficient. Afterward, it was determined whether the final model results were interpreted and explained by explainable AI. Lastly, we checked whether the proposed model is suitable for clinical use or has already been implemented.

## 3 Results

In PubMed, 217 articles were found using the search query mentioned above. Among them, 50 articles met the inclusion criteria based on the title and abstract. In IEEEXplore, 35 articles were found using the search terms, with 11 being considered relevant. Three of these articles were found in PubMed and IEEEXplore. Next, all 44 full texts were checked for eligibility, from which 14 were excluded. Then, the full text of 30 articles from both databases was screened and checked for cross-referencing literature relevant to this review. Of these 83 articles, 21 were found to be potentially relevant.

In total, 51 articles were included in this review. A visual representation of the selection process can be seen in Figure 1, and an overview of the studies is included in Table 2. The upcoming chapters will delve into the various aspects this review aims to elucidate. First, “*data acquisition*” describes how data was obtained, focusing on the frequency of the techniques and the various technical differences. Second, “*age range*” clusters all studies to present an overview of the age ranges that might or might not be covered. Next, the chapter “*Pre-processing*” describes the steps taken after data acquisition and feeding it into the respective model. This chapter follows a structured overview of the diverse “*artificial intelligence models*” found. Then, measures to highlight the “*quality of the models*” will be discussed with the respective findings. Afterward, it will be reported if studies tried to “explain the model’s variables.” Finally, it is explained if authors have opted for “*clinical application*.”

**Table 2:** Overview of the studies included.

References	Study	Dataset	Study population size [%male]	Age range, age mean [SD]
Ball et al. (2017)	Modelling neuroanatomical variation during childhood and adolescence with neighbourhood-preserving embedding.	PING-Study	768 [53%]	3–21 y, 12.3 y
Ball et al. (2021)	Individual variation underlying brain age estimates in typical development	PING-Study	768 [53%]	3–21 y, 12.28 [5.02] y
Ball et al. (2019)	Charting shared developmental trajectories of cortical thickness and structural connectivity in childhood and adolescence	PING-Study	456 [51.1%]	3.2–21.0 y, 12.6 [4.91] y
Brown et al. (2017)	Prediction of brain network age and factors of delayed maturation in very preterm infants	self-recruited	115	27 and 45 w PMA
Brown et al. (2012)	Neuroanatomical assessment of biological maturity	PING-Study	885 [52.2%]	3–20 y, 13.0 [4.9] y
Cao et al. (2015)	Development and validation of a brain maturation index using longitudinal neuroanatomical scans.	National Institute of Health (NIH) pediatric repository	303	4.88–18.35 y
Chen et al. (2022)	Deep learning to predict neonatal and infant brain age from myelination on brain MRI scans.	self-recruited	469	0–25 m GCA, 65.0 [32] w
Chen et al. (2022)			438	0–25 m GCA, 64.4 [30] w
Chen et al. (2022)			389	0–25 m GCA, 61.9 [29]
Chung et al. (2018)	Use of machine learning to determine deviance in neuroanatomical maturity associated with future psychosis in youths at clinically high risk.	PING-Study	953 [51.7%]	3–21 y
Erus et al. (2015)	Imaging patterns of brain development and their relationship to cognition	Philadelphia Neurodevelopmental Cohort	621 [43.5%]	8–22 y, 15.08 y [3.27]
Franke et al. (2012)	Brain maturation: predicting individual BrainAGE in children and adolescents using structural MRI	National Institute of Health (NIH) pediatric repository	394 [52.5%]	5–18 y
Galdi et al. (2020)	Neonatal morphometric similarity mapping for predicting brain age and characterizing neuroanatomic variation associated with preterm birth	Self-recruited	105 [52.3%]	38–45 w
Gschwandtner et al. (2020)	Deep learning for estimation of functional brain maturation from EEG of premature neonates.	Self-recruited	43	24–42 w
He et al. (2020)	Brain age estimation using LSTM on children’s brain MRI	National Institute of Health (NIH) pediatric repository Massachusetts General and Boston Children’s Hospitals	1,212	0–22 y
			428	0–6 y
Hosseinzadeh Kassani et al. (2020)	Causality-based feature fusion for brain neuro-developmental analysis.	Philadelphia Neurodevelopmental Cohort	1,445	8–21 y
Hu et al. (2020)	Hierarchical rough-to-fine model for infant age prediction based on cortical features.	Self-recruited	50	1, 3, 6, 9, 12, 18 and 24 m
Hu et al. (2021)	Accurate brain age prediction model for healthy children and adolescents using 3D-CNN and dimensional attention	ABIDE I ABIDE II ADHD200	880	6–18 y, 11.8y [2.8]
Kardan et al. (2022)	Resting-state functional connectivity identifies individuals and predicts age in 8- to 26-month-olds.	Baby Connectome Project	112 [53.6%]	8–26 m, 15.7 [5.2]
Kawahara et al. (2017)	BrainNetCNN: Convolutional neural networks for brain networks; towards predicting neurodevelopment	Self-recruited	115	24 and 32 m PMA
Kelly et al. (2022)	Investigating brain structural maturation in children and adolescents born very preterm using the brain age framework.	VIBeS PING	768 [52.6%]	3–21 y, 12.28 y,
Khundrakpam et al. (2015)	Prediction of brain maturity based on cortical thickness at different spatial resolutions	National Institute of Health (NIH) pediatric repository	308 [44.2%]	Range not given, 12.9 y [3.8]
Li et al. (2018)	Brain age prediction based on resting-state functional connectivity patterns using convolutional neural networks	Philadelphia Neurodevelopmental Cohort	983	8–22 y
Lund et al. (2022)	Brain age prediction using fMRI network coupling in youths and associations with psychiatric symptoms.	Philadelphia Neurodevelopmental Cohort Healthy Brain Network	1,126	8–22 y
Morita et al. (2022b)	Pediatric brain CT image segmentation methods for effective age prediction models	Self-recruited	204	0–47 m
Morita et al. (2022a)	Quantification of pediatric brain development with X-ray CT images using 3D-CNN	Self-recruited	204	0–47 m
Nielsen et al. (2019)	Evaluating the prediction of brain maturity from functional connectivity after motion artifact denoising.	Self-recruited	122 [54.1%]	7–31 y
O’Toole et al. (2016)	Estimating functional brain maturity in very and extremely preterm neonates using automated analysis of the electroencephalogram	Self-recruited	49	23–32 w GCA
Qu et al. (2020)	BAENET: A brain age estimation network with 3D skipping and Outlier constraint loss	ABIDE II ADHD200 HBN	1915	5–18 y
Saha et al. (2018)	Investigating brain age deviation in preterm infants: a deep learning approach	Self-recruited	86	29–47 w PMA
Shabanian et al. (2019)	Classification of neurodevelopmental age in normal infants using 3D-CNN based on brain MRI	NIMH Data Archive	112	8 d – 3 y
Smyser et al. (2016)	Prediction of brain maturity in infants using machine-learning algorithms.	Self-recruited	50	Preterm: 36–41 w PMA, 38 w [1 w]
			50	Term: 37–41 w PMA, 39 w [1 w]
Stevenson et al. (2017)	Functional maturation in preterm infants measured by serial recording of cortical activity	Self-recruited	43 [41.9%]	25–38 w PMA, 25.6 w
Stevenson et al. (2020)	Reliability and accuracy of EEG interpretation for estimating age in preterm infants.	Self-recruited	62	25–38 weeks PMA
Vandenbosch et al. (2019)	EEG-based age-prediction models as stable and heritable indicators of brain maturational level in children and adolescents.	Self-recruited	Dataset 1: 836	5 and 7 y 16 and 18 y
			Dataset 2: 621	12, 14 and 16 y
Zhao T. et al. (2019)	Unbiased age-specific structural brain atlases for Chinese pediatric population.	Peking University Dataset Beijing HuiLongGuan ADHD200	Dataset 1: 328	6–12 y, 9.03 [1.36]
			Dataset 2: 114	6–12 y, 9.06 [1.38]
			Dataset 3: 71	8–12 y, 10.26 [1.78]
Sturmfels et al. (2018)	A domain guided CNN architecture for predicting age from structural brain images	Philadelphia Neurodevelopmental Cohort	724	8–21 y
Hong et al. (2020)	Brain age prediction of children using routine brain MR images via deep learning	Self-recruited	220	0 to 5 y
Zhao Y. et al. (2019)	Brain age prediction: Cortical and subcortical shape covariation in the developing human brain	Healthy Brain Network Nathan Kline Institute - Rockland Sample	869 (60.9%)	5.02–17.95 y
			210 (58.1%)	6.68–17.94 y
Liang et al. (2019)	Investigating systematic bias in brain age estimation with application to post-traumatic stress disorders	ABIDE CoRR DLBS	566	6 to 89 y
			778	
			315	
Lewis et al. (2018)	T1 white/gray contrast as a predictor of chronological age, and an index of cognitive performance	NIH Pediatric data PING	401	4.5–18.5 y
			760	3–20 y
Dean et al. (2015)	Estimating the age of healthy infants from quantitative myelin water fraction maps	Self-recruited	209 (58.9%)	76–1,526 d
Pardoe and Kuzniecky (2018)	NAPR: a cloud-based framework for neuroanatomical age prediction	ABIDE, ABIDE II, CoRR, DLBS, and NKI Rockland dataset	2,367	Not specified besides figure
Lavanga et al. (2018)	A brain-age model for preterm infants based on functional connectivity.	Self-recruited	30	27–42 w
Liu et al. (2024)	Brain age predicted using graph convolutional neural network explains neurodevelopmental trajectory in preterm neonates	University of California at San Francisco) Benioff Children’s Hospital (UCSF) developing Human Connectome Project	129	32.1–43.4 w
			407 (54.1%)	29–45 w
Tang et al. (2023)	A deep learning-based brain age prediction model for preterm infants via neonatal MRI	Self-recruited	281	27–37 w, 33.4 w
Liu et al. (2023)	Brain age prediction in children aged 0–5 years based on T1 magnetic resonance images	Self-recruited	290	0–5 y
Mendes et al. (2023)	Generalizability of 3D CNN models for age estimation in diverse youth populations using structural MRI.	ABIDE-II ADHD-200 ABCD BHRCS	580	6.1–20.0
			922	7.1–19.9
			11,031	5.8–14.3
			737	8.9–11.1
Zandvoort et al. (2024)	Sensory event-related potential morphology predicts age in premature infants.	research database John Radcliffe Hospital	82	28–40 w PMA
Nielsen et al. (2023)	Maturation of large-scale brain systems over the first month of life.	eLABE CUDDEL O2P2	262	At birth
			45	0–18 m
			5	0-72 h
Hu et al. (2023)	MRI-based brain age prediction model for children under 3 years old using deep residual network.	Self-recruited	658 (62.9%), 230 diseased	0–1,092 d
Griffiths-King et al. (2023)	Predicting ‘Brainage’ in late childhood to adolescence (6–17 yrs) using structural MRI, morphometric similarity, and machine learning.	Autism Brain Imaging Data Exchange cohort from the Pre-Procecessed Connectome Project	327 (79.2%)	6.5–16.9,12.4 [± 2.5]
Bellantuono et al. (2021)	Predicting brain age with complex networks: From adolescence to adulthood	ABIDE	1,112	7 to 64 y

Study population size is presented in total and the percentage of males, if given. Age range is further broken down to mean and standard deviation (SD), if presented by authors. Years (y), months (m), weeks (w), post menstrual age (PMA), gestation corrected age (GCA).

### 3.1 Data acquisition

Several authors used publicly available datasets, including the Pediatric Imaging, Neurocognition, and Genetics (PING) Data Repository (Di Martino et al., 2014), ABIDE I & II (Di Martino et al., 2014; Di Martino et al., 2017), ADHD2000 (Milham et al., 2012), National Institutes of Health Pediatric Repository (NIH-PR) (Evans, 2006), Autism Brain Imaging Data Exchange cohort from the Pre-Processed Connectome Project (Bellec, 2017), Healthy Brain Network (Alexander et al., 2017), The NKI Rockland Sample (2024), consortium for reliability and reproducibility study (CoRR) (Zuo et al., 2014), The Developing Human Connectome Project (2024), Adolescent Brain Cognitive Development-Study (ABCD) (Scientists, 2024), Brazilian High Risk Cohort Study (BHRC) (Simioni et al., 2017), eLABE (Stout et al., 2022), CUDDEL (Rogers and Agrawal, 2024) and *Philadelphia Neurodevelopmental Cohort* (Satterthwaite et al., 2016).

As for the acquisition modality, most of the literature in this review used magnetic resonance imaging (MRI) for data acquisition (44/51) (Ball et al., 2017, 2019, 2021; Bellantuono et al., 2021; Brown et al., 2012, 2017; Cao et al., 2015; Chen et al., 2022; Chung et al., 2018; Dean et al., 2015; Erus et al., 2015; Franke et al., 2012; Galdi et al., 2020; Griffiths-King et al., 2023; He et al., 2020; Hong et al., 2020; Hosseinzadeh Kassani et al., 2020; Hu et al., 2020, 2021, 2023; Tang et al., 2023; Kardan et al., 2022; Kawahara et al., 2017; Kelly et al., 2022; Khundrakpam et al., 2015; Lewis et al., 2018; Li et al., 2018, 2020; Liang et al., 2019; Liao et al., 2020; Liu et al., 2023, 2024; Lund et al., 2022; Mendes et al., 2023; Nielsen et al., 2019, 2023; Pardoe and Kuzniecky, 2018; Qu et al., 2020; Saha et al., 2018; Shabanian et al., 2019; Smyser et al., 2016; Sturmfels et al., 2018; Zhao T. et al., 2019; Zhao Y. et al., 2019). The 44 studies using MRI can be further subdivided by the strength of the magnetic field, the weighting of structural MRI, functional MRI, and diffusion MRI. Twenty-three studies used solely T1-weighted MRI for structural MRI (Ball et al., 2017, 2021; Bellantuono et al., 2021; Cao et al., 2015; Chung et al., 2018; Franke et al., 2012; Griffiths-King et al., 2023; He et al., 2020; Hong et al., 2020; Hu et al., 2021, 2023; Tang et al., 2023; Kelly et al., 2022; Khundrakpam et al., 2015; Lewis et al., 2018; Liang et al., 2019; Liu et al., 2023, 2024; Mendes et al., 2023; Pardoe and Kuzniecky, 2018; Qu et al., 2020; Sturmfels et al., 2018; Zhao Y. et al., 2019). Three studies undertook a combined approach of T1- and T2-weighted MRI (Chen et al., 2022; Hu et al., 2020; Zhao T. et al., 2019). Shabanian et al. (2019) added a proton density MRI to the T1- and T2-weighted MRI. Two studies solely relied on resting-state functional MRI (rs-fMRI) (Hosseinzadeh Kassani et al., 2020; Li et al., 2018). Further, only one group used diffusion MRI (Saha et al., 2018) or diffusion tensor imaging (Kawahara et al., 2017). Four authors combined structural MRI with diffusion MRI (Ball et al., 2019; Brown et al., 2012, 2017; Galdi et al., 2020), six with rs-fMRI (Kardan et al., 2022; Li et al., 2020; Lund et al., 2022; Nielsen et al., 2019, 2023; Smyser et al., 2016), one with proton density (Khundrakpam et al., 2015), and another two with diffusion tensor imaging (Brown et al., 2012; Erus et al., 2015). Once, T2-weighted MRI was combined with resting-state fMRI (Nielsen et al., 2023).

The MRI field strength was 1.5 T and 3 T and was used by sixteen (Ball et al., 2017, 2019, 2021; Brown et al., 2012; Chung et al., 2018; Erus et al., 2015; Galdi et al., 2020; Hong et al., 2020; Hu et al., 2020; Kardan et al., 2022; Kelly et al., 2022; Li et al., 2020; Liu et al., 2023; Lund et al., 2022; Nielsen et al., 2019; Zhao T. et al., 2019) and thirteen (Brown et al., 2017; Cao et al., 2015; Franke et al., 2012; Griffiths-King et al., 2023; He et al., 2020; Hosseinzadeh Kassani et al., 2020; Hu et al., 2023; Khundrakpam et al., 2015; Li et al., 2018; Liang et al., 2019; Nielsen et al., 2023; Shabanian et al., 2019; Sturmfels et al., 2018) studies, respectively. Ten studies used 3 and 1.5 T (Hu et al., 2021, 2023; Lewis et al., 2018; Liu et al., 2024; Mendes et al., 2023; Pardoe and Kuzniecky, 2018; Qu et al., 2020; Zhao Y. et al., 2019). Six studies did not mention the field strength of the utilized MRI scanner (Chen et al., 2022; Dean et al., 2015; Tang et al., 2023; Kawahara et al., 2017; Saha et al., 2018; Smyser et al., 2016). 15 studies used 3D MRI data via Magnetization Prepared – RApid Gradient Echo (MPRAGE), 3D-RF-spoiled gradient echo sequence, or other procedures (Ball et al., 2017, 2019, 2021; Brown et al., 2012; Cao et al., 2015; Chung et al., 2018; Erus et al., 2015; Franke et al., 2012; Galdi et al., 2020; He et al., 2020; Hu et al., 2021; Khundrakpam et al., 2015; Lewis et al., 2018; Liu et al., 2024; Lund et al., 2022; Nielsen et al., 2019; Qu et al., 2020; Sturmfels et al., 2018; Zhao T. et al., 2019). Chen et al. (2022) mixed their data, with 57.6% 3D and the remainder 2D. Khundrakpam et al. (2015) used 3D T1-weighted, but 2D T2-weighted MRI. Hong et al. (2020) relied solely on 2D images. The remaining studies mixed datasets, which led to heterogeneous inputs.

The second most common data acquisition type for model training was via EEG, which was used by seven studies (Gschwandtner et al., 2020; O’Toole et al., 2016; Stevenson et al., 2017, 2020; Vandenbosch et al., 2019; Lavanga et al., 2018; Zandvoort et al., 2024). The number of electrodes used in these studies varied. Vandenbosch et al. (2019) used the most electrodes, thirty, whereas Gschwandtner et al. (2020) used eight to two electrodes. From the remaining studies, Stevenson et al. (2017, 2020) and Lavanga et al. (2018) used nine electrodes, and O’Toole et al. (2016) used ten. Zandvoort et al. (2024) combined EEG with electromyography (EMG) and used a 64-channel headbox. Only two publications, Morita et al. (2022a,b), used CT scan images for model creation.

### 3.2 Age range

It must be noted that the reporting age structure is not standardized throughout the literature. Some authors stated the age range, whereas others reported the mean age, including standard deviation. These studies specified that these datasets were predominantly chosen due to the high number of images they provided. However, the authors checked the images of the datasets manually for their respective inclusion criteria, which explains the usage of the same dataset in various studies but varying age ranges. In total, 28 studies relied on publicly available datasets (Ball et al., 2017, 2019, 2021; Bellantuono et al., 2021; Brown et al., 2012; Cao et al., 2015; Chung et al., 2018; Erus et al., 2015; Franke et al., 2012; Griffiths-King et al., 2023; He et al., 2020; Hosseinzadeh Kassani et al., 2020; Hu et al., 2021; Kardan et al., 2022; Kelly et al., 2022; Khundrakpam et al., 2015; Lewis et al., 2018; Li et al., 2018; Liang et al., 2019; Liu et al., 2024; Lund et al., 2022; Mendes et al., 2023; Nielsen et al., 2023; Pardoe and Kuzniecky, 2018; Qu et al., 2020; Shabanian et al., 2019; Sturmfels et al., 2018; Zhao T. et al., 2019). The total covered age ranges from 0 to 89 years.

Twenty three studies used newborns in their early weeks or up to three years (Brown et al., 2017; Chen et al., 2022; Dean et al., 2015; Galdi et al., 2020; Hu et al., 2020, 2023; Tang et al., 2023; Kardan et al., 2022; Kawahara et al., 2017; Li et al., 2020; Liu et al., 2024; Nielsen et al., 2023; Saha et al., 2018; Shabanian et al., 2019; Smyser et al., 2016; Gschwandtner et al., 2020; O’Toole et al., 2016; Stevenson et al., 2017, 2020; Lavanga et al., 2018; Zandvoort et al., 2024; Morita et al., 2022a,b). From these, 12 authors included preterm infants (Brown et al., 2017; Galdi et al., 2020; Tang et al., 2023; Liu et al., 2024; Saha et al., 2018; Smyser et al., 2016; Gschwandtner et al., 2020; O’Toole et al., 2016; Stevenson et al., 2017, 2020; Lavanga et al., 2018; Zandvoort et al., 2024). Two studies created a Model that included the age range of 0–5 years (Hong et al., 2020; Liu et al., 2023).

Another cluster of 25 studies took images starting in the age range of older than 3 to 89 years (Ball et al., 2017, 2019, 2021; Bellantuono et al., 2021; Brown et al., 2012; Cao et al., 2015; Chung et al., 2018; Erus et al., 2015; Franke et al., 2012; Griffiths-King et al., 2023; Hosseinzadeh Kassani et al., 2020; Hu et al., 2021; Kelly et al., 2022; Khundrakpam et al., 2015; Lewis et al., 2018; Li et al., 2018; Liang et al., 2019; Lund et al., 2022; Mendes et al., 2023; Nielsen et al., 2019; Qu et al., 2020; Sturmfels et al., 2018; Zhao T. et al., 2019; Zhao Y. et al., 2019; Vandenbosch et al., 2019).

**Figure 2:**
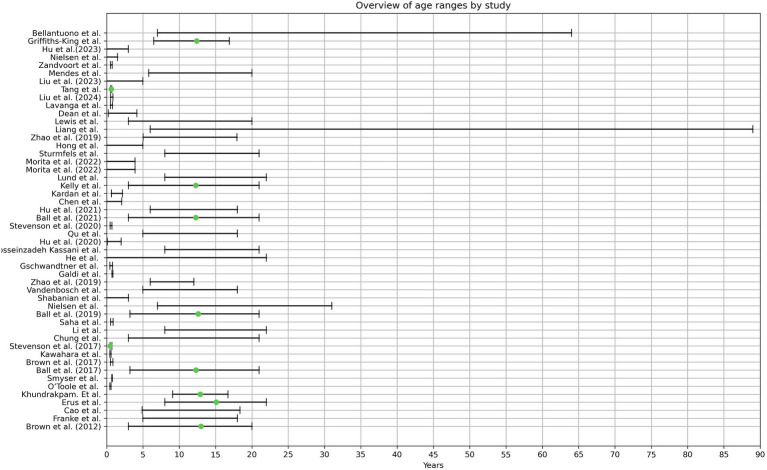
The graphical illustration depicts the age ranges observed in the analyzed literature. Pardoe et al. were excluded from this representation due to unclearly stated age ranges. The green dot denotes the average age, as provided by the respective authors.

Only the publication from He et al. (2020) covered the age range from 0–22 years, thus being the only study that included new-born children until late adolescence and adulthood. The age ranges per study can be seen in Figure 2.

### 3.3 Pre-processing

In this review, different acquisition methods were found and included. Therefore, this section compares the processing steps and illustrates a typical pipeline per acquisition method. Pre-processing starts after acquiring the data, which excludes data correction applied by the device, e.g., motion correction by MRI systems, and ends with the data being fed into the AI model.

**Figure 3:**
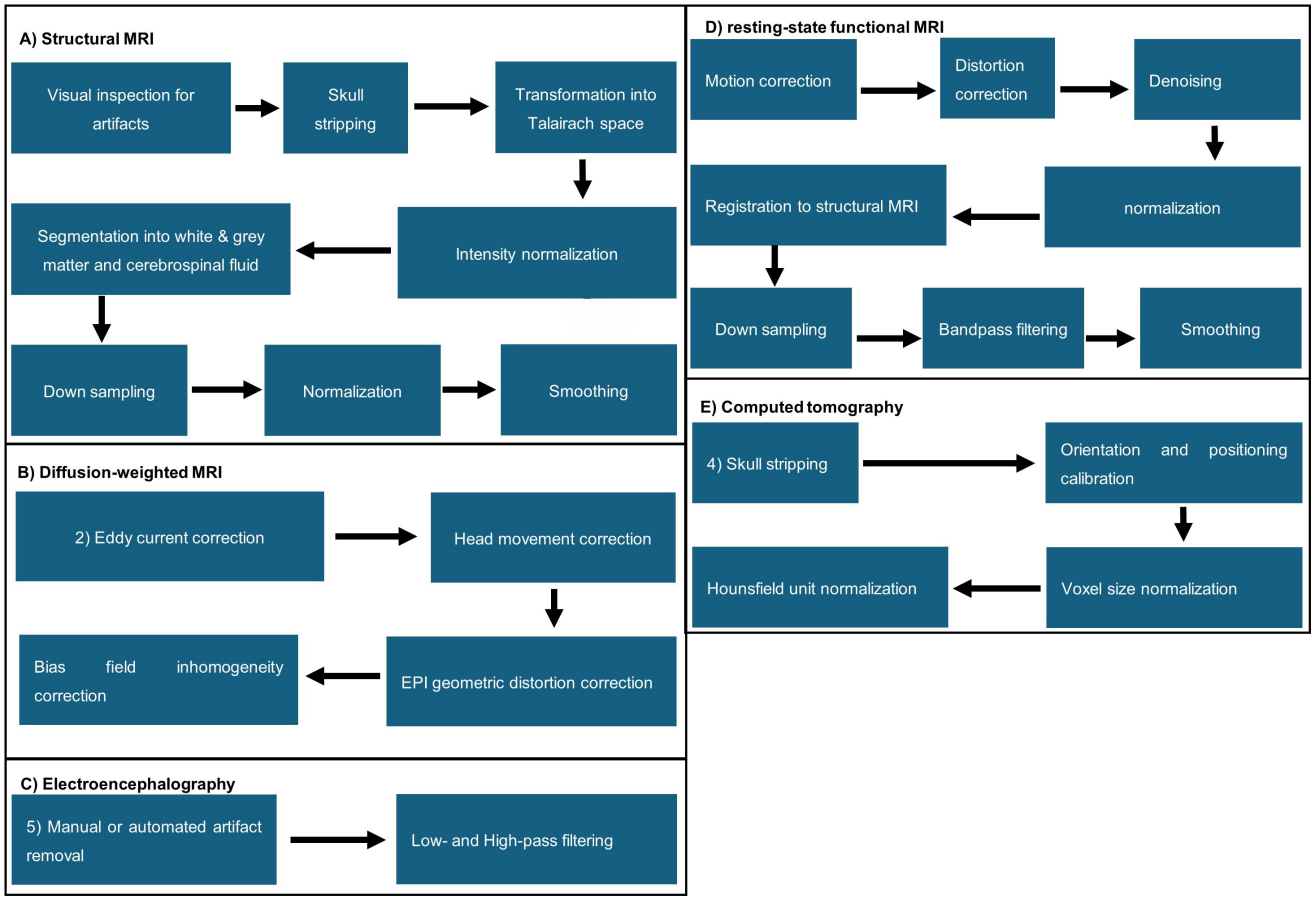
Visual representation of MRI the commonly applied pre-processing steps found in the analyzed literature. (A) Structural MRI; (B) diffusion-weighted MRI; (C) electroencephalography; (D) resting-state functional MRI; (E) computed tomography.

Handling of structural MRI data follows similar pipelines, but the sequence of individual steps differs throughout the literature. Generally, a separate visual inspection for artifacts was the first step, followed by transformation from DICOM to NIFTI and skull stripping, performed manually or by algorithms, e.g., FreeSurfer, Statistical Parametric Mapping (SPM12, SPM8, or DATEL), CAT12 toolbox, CIVET (BIC, 2024), Brain Extraction Tool, or LABEL (Shi et al., 2012; Fischl, 2012; SPM, 2023; Penny et al., 2006; Gaser et al., 2024; Smith, 2002). Next, the images were transformed into Talairach space, followed by intensity normalization. Further, the brain was segmented into the white and grey matter and cerebrospinal fluid via FreeSurfer (Zhao T. et al., 2019; O’Toole et al., 2016), LABEL (Sturmfels et al., 2018), Statistical Parametric Mapping (SPM12, SPM8, or DATEL) (Penny et al., 2006), and FSL’s fast (Stevenson et al., 2017). The white and grey matter boundaries of the brain were further tessellated. Lastly, data was often downsampled, normalized, and smoothed. A visual representation of the preprocessing steps is illustrated in Figure 3. Hong et al. was the only MRI-based study that did not perform skull stripping while using structural MRI scans for their data. Additionally, they used data augmentation to increase the dataset artificially. They split the MRI scans into slices, which were then transformed via scaling, rotation, translation, and gamma correction. The resulting slices were then stacked again (Hong et al., 2020). Shabanian et al.’s Figure 3 shows segmented data but does not describe how segmentation was performed. We could not determine if the dataset was already preprocessed from the resources the authors mentioned (Shabanian et al., 2019). Regarding diffusion-weighted MRI/diffusion tensor imaging, eddy current, head movement, and EPI geometric distortions, bias field inhomogeneity correction and intensity scaling correction were applied. Voxel-wise maps were derived and co-registered to the template space from the fractional anisotropy and apparent diffusion coefficient. Registration to T1- or T2-Space, probabilistic whole-brain fiber tracking, and alignment were processed by FSL Diffusion Toolbox (FSL, 2024), MRtrix 3.0 (MRtrix3, 2024) or manually. Resting-state functional MRI pre-processing steps included motion correction, distortion correction, registration to the native T1- or T2-weighted MRI, normalization, denoising, down-sampling of spatial information, bandpass filtering, and smoothing. This was sometimes followed by z-transformation of the estimated correlations to Fisher’s transformation to create a correlation matrix (Lund et al., 2022; Nielsen et al., 2019; Smyser et al., 2016).

In CT images, skull stripping, orientation and position calibration, voxel size normalization, and normalization of CT values were performed. Morita et al. (2022b) tried prediction without previous segmentation but failed with this approach. In contrast, Hu et al. (2023) used ResNet-18 and found that their model could predict chronological age from raw and preprocessed MRI data. A preprocessing guideline for diffusion tensor imaging was not found.

EEG datasets were always filtered with a bandpass filter. Low and high cut-off frequencies varied from 0.5 Hz to 64 Hz, respectively. Some authors removed artifacts via individual inspection or algorithms, e.g., the FBA algorithm (Stevenson et al., 2017) and PureEEG (Stevenson et al., 2020; Lund et al., 2022; Nielsen et al., 2023; Pardoe and Kuzniecky, 2018). Lavanga et al. analyzed sleep stages (quiet vs. non-quiet sleep) that were marked manually. In addition, specific patterns, such as spontaneous activity that indicates deep sleep patterns in newborns, were automatically annotated and segmented using SAT detection algorithms.

### 3.4 Artificial intelligence models

The preferred AI model used by 22 teams is the kernel-based learning algorithm (Brown et al., 2017; Chung et al., 2018; Erus et al., 2015; Hosseinzadeh Kassani et al., 2020; Kardan et al., 2022; Zhao T. et al., 2019; O’Toole et al., 2016; Stevenson et al., 2017, 2020; Nielsen et al., 2019, 2023; Smyser et al., 2016; Vandenbosch et al., 2019; Galdi et al., 2020; Franke et al., 2012; Zhao Y. et al., 2019; Zandvoort et al., 2024; Liang et al., 2019; Griffiths-King et al., 2023; Pardoe and Kuzniecky, 2018; Liu et al., 2023). These are five support vector machines (SVM) (Galdi et al., 2020; Hosseinzadeh Kassani et al., 2020; Smyser et al., 2016; Stevenson et al., 2020; Vandenbosch et al., 2019), 12 support vector regressions (SVR) (Brown et al., 2017; Chung et al., 2018; Erus et al., 2015; Kardan et al., 2022; Liang et al., 2019; Liu et al., 2023; Nielsen et al., 2019, 2023; Zhao T. et al., 2019; O’Toole et al., 2016; Stevenson et al., 2017; Zandvoort et al., 2024), and six Relevance Vector Machines (RVM) (Franke et al., 2012; Griffiths-King et al., 2023; Pardoe and Kuzniecky, 2018; Zhao T. et al., 2019; Vandenbosch et al., 2019). Galdi et al. (2020) and Smyser et al. (2016) used a support vector machine to classify term-born and preterm-born. Zhao et al. used SVM to show that the atlas choice is critical to improving brain age prediction. There might be differences in brain development if children differ in ethnicity or grow up in different countries (Zhao T. et al., 2019). Pardoe et al. found that the relevance vector regression outperformed the tested Gaussian process regression. Ten of 22 studies used a linear kernel for kernel selection, making it the predominantly used type (Erus et al., 2015; Galdi et al., 2020; Hosseinzadeh Kassani et al., 2020; Kardan et al., 2022; Liang et al., 2019; Nielsen et al., 2023; Smyser et al., 2016; Zhao T. et al., 2019; O’Toole et al., 2016; Zandvoort et al., 2024). Liu et al. (2023) and Chung et al. (2018) used a radial basis function kernel. Griffiths-King et al. compared a Laplace radial basis function kernel to a Gaussian radial basis function. They found the first superior in their relevance vector and Gaussian Process Regression (Griffiths-King et al., 2023). A smoothing kernel was only used by Franke et al. (2012). Seven studies did not further specify which kernel was used (Brown et al., 2017; Griffiths-King et al., 2023; Nielsen et al., 2019; Pardoe and Kuzniecky, 2018; Stevenson et al., 2017, 2020; Vandenbosch et al., 2019).

The second most frequently used model type in this literature review is the convolutional neural network (CNN), which has been subject to 19 studies (Bellantuono et al., 2021; Chen et al., 2022; Hong et al., 2020; Hu et al., 2021, 2023; Tang et al., 2023; Kawahara et al., 2017; Li et al., 2018; Liang et al., 2019; Liu et al., 2024; Mendes et al., 2023; Qu et al., 2020; Saha et al., 2018; Shabanian et al., 2019; Sturmfels et al., 2018; Gschwandtner et al., 2020; Morita et al., 2022a,b). For structural MRI Input, eleven studies created a 3D-CNN that differed in architectures (Chen et al., 2022; Hong et al., 2020; Hu et al., 2021, 2023; Tang et al., 2023; Mendes et al., 2023; Qu et al., 2020; Shabanian et al., 2019; Sturmfels et al., 2018; Morita et al., 2022a,b), and four used a 2D-CNN (He et al., 2020; Li et al., 2018; Saha et al., 2018; Gschwandtner et al., 2020). Bellantuono et al. (2021) used a feedforward deep neural network implemented with the “h2o” R package (H2O.ai, 2024). In one of their studies, Liu et al. (2024) present a novel approach called a “graph convolutional neural network.”

Various approaches were employed in studies on age prediction using 2D CNNs. Saha et al. (2018) utilized a 19-layer 2D CNN model and trained their model from patches taken from MRI scans instead of full scans. Li et al. (2018) employed a 21-layer deep CNN with residual blocks. Gschwandtner et al. (2020) developed three 14-layer CNN models for different EEG electrode configurations. He et al. compared their method to a 3D CNN by utilizing a ResNet-18 combined with a bidirectional LSTM for age prediction. They found that the proposed 2D CNN-LSTM method outperformed the 3D CNN (He et al., 2020).

The 3D CNNs were structured very heterogeneously. The number of layers varied from 10 to 32. Tang et al. used a network called BAPNET, based on the Inception-Resnet-v2 framework, and compared a 2D to 3D approach. They found that the 3D model had a higher MAE, and the R^2^ was better than the 2D model (Tang et al., 2023). Shabanian et al. also implemented a 4-block architecture and compared 2D and 3D models. Upon comparing their 3D-CNN with a similar 2D-CNN, they discovered that their 3D-CNN achieved the same accuracy in just 14 epochs, in contrast to the 2D-CNN, which required over 200 epochs (Shabanian et al., 2019). Hong et al. also compared a 2D to a 3D CNN. Their data was composed of MRI slices with gaps between adjacent slices. While the 2D CNN was thought to perform equally well, the 3D CNN performance difference was significant. This suggests that 3D CNN might outperform 2D CNN in tasks for stacked 2-dimensional data (Hong et al., 2020). Kawahara et al. adapted their model to the acquired diffusion tensor imaging brain network data. They introduced three additional layers to their CNN that are thought to implement topological differences between images and brain network data. Namely, these are edge-to-edge, edge-to-node, and node-to-graph layers. These layers incorporate extra convolutional filters and carry out specific operations on the brain network to extract features. The filters amalgamate all feature maps from the previous output layer and generate a new output for the subsequent layer (Kawahara et al., 2017). Chen et al. used a 3D regression CNN for deep learning with 27 layers and Adam optimization to predict the gestational corrected age in neonates and infants (Chen et al., 2022; Kingma and Ba, 2017). Qu et al. have introduced a brain age estimation network featuring 3D skipping and outlier constraint loss. The network, comprising 25 layers, is structured into feature extraction and combination. The feature extraction component consists of 4 blocks, each housing a 3D convolution layer with leaky ReLU, a subsequent 3D convolution layer with leaky rectified linear unit (ReLU) and group normalization, and a max pooling layer. Referring to ResNet (He et al., 2016), the authors have implemented a connection between the first and last layer, effectively skipping the second convolution layer (Qu et al., 2020; He et al., 2016). The feature combination consists of 3 convolutional layers. The novelty the authors describe is the combination of MSE loss and Huber loss as a loss function. Combining these two functions increases the robustness while maintaining the model-fitting ability and decreasing the gradient changes for outliers (Qu et al., 2020). Sturmfels et al. (2018) compared their model to the architecture of Cole et al. (2017) and modified it by decreasing the number of filters after each convolutional block instead of increasing these and segmenting the brain into eight separate regions. These changes are considered easy to implement and improve time and age prediction (Sturmfels et al., 2018).

Hu et al. (2021) propose a dimensional-attention-based 3D CNN (DACNN), which can be structured in three parts. First, the down-sampling of the MRI images reduces the image size. Second, feature extraction of deep nonlinear features by four blocks. Third, feature combination and age prediction. The dimensional attention block is a novelty, used in the down-sampling and feature extraction parts. It suppresses noise while highlighting more important parts without altering the semantics or shape of the input data (Hu et al., 2021). Another Block the team introduced is called the diluted residual block and is based on the 3D residual blocks from ResNet and introduced by Yu et al. (2017). The training took 100 epochs with the Adam optimizer and a mean square error loss function (Hu et al., 2021). In 2023, Hu et al. built a model using the modified ResNet-18 from He et al. and tried different inputs. The model outperformed the compared SVR and could predict age accurately from segmented and raw data (Hu et al., 2023; He et al., 2015).

Morita et al. published two articles in 2022 using the same 3D CNN on computed tomography data. The model consists of 23 layers, and training took 600 epochs (Morita et al., 2022a,b). According to the authors, model training proved unsuccessful without cranial segmentation (Morita et al., 2022b). Liu et al. propose a Graph convolutional neural network (GCN). They created a cortical mesh and formed a sparse binary adjacency matrix. Further, sulcal depth, cortical thickness, and the grey/white matter ratio were given to the GCN as harmonized graphs. The graphs’ vertices were rearranged via 1D pooling. In total, three pooling and three convolutional layers were used. The Model used 700 epochs for training. According to the author, the GCN outperformed the contested morphometry-based CNN (Liu et al., 2024).

The third most used model was the Gaussian Process Regression (GPR), used in eight articles (Ball et al., 2017, 2019, 2021; Griffiths-King et al., 2023; Kelly et al., 2022; Liang et al., 2019; Liu et al., 2023; Pardoe and Kuzniecky, 2018). In their 2021 study, Ball et al. developed a range of machine learning (ML) models. They determined that Gaussian Process Regression (GPR) performed comparably to regularized linear regression with elastic net and outperformed gradient-boosted regression trees (Ball et al., 2021). Griffiths-King et al. (2023) discovered that GPR outperformed the relevance vector regression model, while Pardoe and Kuzniecky (2018) concluded that the RVR was superior. Liu et al. (2023) tested RVR, GPR, SVR, Random Forest Regression (RFR), and K-neighborhood regression, concluding that RFR and GPR performed superior. Brown et al. (2017), Khundrakpam et al. (2015), Lavanga et al. (2018), and Lund et al. (2022) used a linear regression model.

The teams Brown et al. (2017), Liu et al. (2023), and Vandenbosch et al. (2019) used random forests. Brown et al. (2017) computed five different models and concluded that random forests worked best. The remaining models were a linear regression model, a multi-layer perceptron, SVR, and a bagging regression (Brown et al., 2017). Vandenbosch et al. also showed that random forests worked best for age prediction and age classification (puberty/adolescent) compared to SVM and RVM. Although their random forest model yielded the lowest mean predicted error (MPE), the authors noted that it cannot not predict continuous numbers, thus scoring better compared to relevance vector machines (Vandenbosch et al., 2019).

Two authors used penalized ridge regression (Liang et al., 2019; Zhao Y. et al., 2019). Liang et al. compared multiple models and showed deep neural networks and Gaussian Process Regression outperform penalized ridge regression. However, every model overestimated the age in children and underestimated it in adults (Liang et al., 2019). Zhao Y. et al. (2019) were the only authors that created only one ridge regression model and compared its performance on two separate datasets.

Three studies used a regularized linear regression with elastic net penalty (Ball et al., 2021; Galdi et al., 2020; Lewis et al., 2018). Moreover, only one study used LASSO as a multivariate linear regression as their Model design (Cao et al., 2015). Brown et al. (2012) used a regularized multivariate nonlinear regression-like approach that used a set of pre-chosen variables found in the literature. The authors created four different statistical models. One model was fed with all imaging modalities, whereas the remaining three were only fed with T1-, T2-, or diffusion MRI-derived measures. The overall set of variables was then selected based on evidence from the literature. Next, these predictors were regularized using rotation, orthogonalization, and normalization, relying on the Mahalanobis distance technique, followed by a whitening transformation for decorrelation. Further, the shrinkage technique was applied to circumvent possible overfitting (Brown et al., 2012).

Hu et al. was the only group applying a Hierarchical Rough-to-Fine (HRtoF) model to age prediction. First, a rough prediction stage, in which a Bayesian linear discriminant classifies a rough age group. Second, a fine prediction stage follows, in which each age group has its own linear regression model to further narrow down the exact age group. In addition, a conventional one-stage prediction model was applied when the scan could not properly be assigned to any subgroup (Hu et al., 2020).

**Figure 4:**
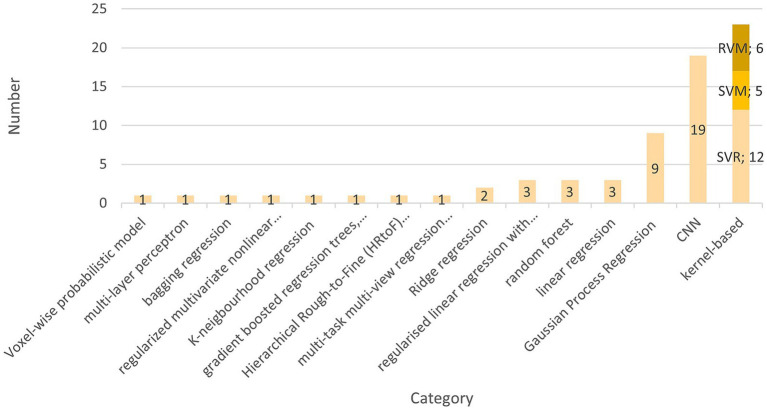
This figure shows the cumulative number of AI methods found in the literature. Kernel-based methods are subdivided into support vector regressions, support vector machines and relevance vector regressions.

Dean et al. used myelin water fraction maps for a voxel-wise probabilistic model. These myelin-water-fraction maps reflect the amount of water trapped in the myelin, which can be used to determine age as the amount of myelin increases with higher age (Dean et al., 2015). Figure 4 presents an overview of the AIs used and their respective frequency.

### 3.5 Quality

The quality of the models is described very heterogeneously because models are assessed differently depending on the aim of the study and the method used. However, most studies preferred to present their models via mean absolute error (MAE). Some authors also stated the root mean squared error (RMSE), standard deviation (SD), correlation coefficient (r), coefficient of determination (r^2^), F1-score, AUC, or precision. Given the diverse age range and various AI approaches, the best model for age prediction cannot be determined, but studies can be clustered and compared within the same group. All models depend on the information they receive, thus leaving the dataset as the most prominent feature that can be used for proper comparison. However, this does not mean that self-recruiting studies cannot be assessed. This review does not compare models based on the underlying machine learning models but rather on the datasets that have been used. An overview can be found in Table 3.

**Table 3:** This table represents an overview of artificial intelligences, its respective performance, and data acquisition of each study.

References	AI	Resolution	Data acquisition	MAE [SD], r^2^
Ball et al. (2017)	Gaussian Process Regression	Not stated	3 T, T1- and T2-weighted MRI & diffusion weighted scans	1.54 y, r = 0.926
Ball et al. (2021)	Regularized linear regression with elastic net penalty	Not stated	3 T, T1-weighted MRI images	1.81 y [0.06], 0.79
	Gaussian process regression			1.75 y [0.07], 0.81
	Ensemble model			1.92 y [0.10], 0.78
Ball et al. (2019)	Gaussian Process Regression	Image resolution was 1 × 1 × 1.2 mm (Siemens) or 0.9375 × 0.9375 × 1.2 mm (GE) Diffusion data: resolution 2.5 × 2.5 mm (Siemens) or 1.875 × 1.875 (GE) and slice thickness = 2.5 mm	3 T T1-weighted MRI & diffusion MRI	2.18 y [0.18]
Brown et al. (2017)	Linear regression	0.625 mm × 0.625 mm × 3 mm (Brown et al., 2015)	1.5 T T1-weighted MRI & diffusion MRI scan	6.284 w [4.230 SDAE]
	Multi-layer perceptron			7.223 w [5.080 SDAE]
	SVR			1.712 w [1.366 SDAE]
	Bagging regression			1.559 w [1.255 SDAE]
	Random forests			1.554 w [1.197 SDAE]
Brown et al. (2012)	Regularised multivariate nonlinear regression-like	T1: slice thickness = 1.2 mm T2: slice thickness = 2.5 mm	3 T, T1- and T2-weighted MRI & diffusion weighted scans	1.03 y, 0.92
Cao et al. (2015)	LASSO/multivariate linear regression	GE scanners: slice thickness, 1.5 mm Siemens scanners: slice thickness, 1 mm	1.5 T T1-weighted MRI	1.69 y, r = 0.82
Chen et al. (2022)	3DCNN using T1 & T2	Not stated	T1-, T2-weighted MRI	7.7 w [1.7]
	3DCNN using T1			9.8 w [2.3]
	3DCNN using T2			9.1 w [1.9]
Chung et al. (2018)	Support vector regression with radial basis function kernel	1.2-mm slices (256 × 192-mm) in-plane resolution (PING study)	3 T T1-MRI weighted	1.69 y, 0.84
Erus et al. (2015)	SVR with a linear kernel	0.9375 mm × 0.9375 mm × 1 mm	3 T T1-MRI weighted & Diffusion Tensor Imaging MRI	1.22 y
Franke et al. (2012)	RVR with a smoothing kernel	1 × 1 × 1 mm^3^ 1.5 mm or 1 mm (Siemens) slice thickness	1.5 T, T1-MRI-weighted	1.1 y, r = 0.93
Galdi et al. (2020)	Linear regression model with elastic net regularisation	acquired voxel size = 1 mm isotropic T1 & T2	3 T T1 MRI-weighted & diffusion MRI	0.7 w [0.56], r = 0.78
Gschwandtner et al. (2020)	CNN	n.a.	EEG (8, 4 & 2 electrodes)	8 EEG electrodes: 93.6% of estimations lying within ±2 w 67.9% within ±1 w deviation from PMA
He et al. (2020)	CNN	NIH-PD and MGHBCH	1.5 T, T1-MRI	0.96 y
Hosseinzadeh Kassani et al. (2020)	SVM classification with linear kernel	voxel size = 3 mm^3^	Resting-state-functional MRI	0.929 y [0.041]
Hu et al. (2020)	Hierarchical Rough-to-Fine model	T1: resolution with 1 × 1 × 1 mm^3^ T2: 1.25 × 1.25 × 1.95 mm^3^	3 T, T1-, T2-weighted MRI	32.1 [1.2 days]
Hu et al. (2021)	Dimensional-attention-based 3D convolutional neural network	ABIDE I & II, ADHD200	3 T, T1-MRI	1.01 y, MSE: 1.92y, 0.73
Kardan et al. (2022)	SVR with a linear kernel	T1 & T2 slice thickness 0.8 mm	3 T, T1- T2 MRI & rs-fMRI	3.6 m, 0.51
Kawahara et al. (2017)	CNN	Not stated	diffusion tensor imaging	2.17 w [1.59]
Kelly et al. (2022)	Gaussian process regression	Victorian Infant Brain Study (VIBeS)	3 T T1 MRI	1.72 y [0.16], 0.80
Khundrakpam et al. (2015)	Linear regression	1 mm isotropic data GE scanner – 1.5 mm Fallback protocol 3 mm	1.5 T, T1-MRI, T2-MRI, Proton Density	1.68 y, r = 0.84
Li et al. (2018)	CNN	Philadelphia Neurodevelopmental Cohort (PNC)	Rs-fMRI	2.15y [1.54] R0.4 = 0.614
Lund et al. (2022)	Linear regression	Healthy Brain Network (HBN) study sample & Philadelphia Neurodevelopmental Cohort (PNC)	3 T T1 MRI & T2 rs-fMRI	2.43 y [2.93], r = 0.6
Morita et al. (2022b)	3DCNN	n.a.	CT	4.61 m [3.65], r = 0.89
Morita et al. (2022a)	3DCNN	n.a.	CT	RMSE: 6.45 m R = 0.89 Mean Prediction Error: 2.29 m [6.04]
Nielsen et al. (2019)	SVR	T1: 1 × 1 × 1 mm^3^ voxels	3 T T1 MRI & rs-fMRI	R2 = 0.57
O’Toole et al. (2016)	SVR with a linear kernel	n.a.	EEG (10 electrodes)	[7.85] d, r = 0.889
Qu et al. (2020)	3DCNN	ABIDE, ADHD200, HBN resampled to 1.5 × 1.5 × 1.5 mm^3^	T1 MRI	1.11, 0.78
Saha et al. (2018)	2DCNN	field of view 224 × 224 mm, matrix 128 × 128 in plane res: 1.75 × 1.75 mm, slice thickness not given	Diffusion MRI	R = 0.6
Shabanian et al. (2019)	3DCNN	NIMH Data Archive (NDA)	1.5 T, T1- T2-MRI & Proton density MRI	Class, Precision, F1-Score New-born, 1.00, 1.00; 1 Year, 0.95, 0.97; 3 Years, 1.00, 0.99
Smyser et al. (2016)	SVM with a linear kernel	voxel size 1 × 1 × 1 mm^3^	T2-MRI, rs-fMRI	Preterm vs. Term classification: 84% accuracy, 90% sensitivity and 78% specificity
Stevenson et al. (2017)	SVR	n.a.	EEG (9 electrodes)	R = 0.936
Stevenson et al. (2020)	SVM	n.a.	EEG (9 electrodes)	Random error = 1.1 w Systematic error = −0.1 w
Vandenbosch et al. (2019)	Random Forest	n.a.	EEG (30 electrodes)	1.22 y
	RVM			1.46 y
	SVM			Not presented
Zhao T. et al. (2019)	SVM with a linear kernel	ADHD-200 T1: acquisition matrix: 256 × 256, FOV: 256 × 256 mm^2^; slice thickness – slice thickness 1.33 mm 1 × 1 × 1.33 mm Beijing cohort T1: in-plane resolution 1.0 × 1.0 mm, slice thickness 1.0 mm T2: in-plane resolution—0.7 × 0.7 mm, slice thickness—0.7 mm,	3 T, T1- T2-MRI	r = 0.48
	RVM with a linear kernel			r = 0.48
Sturmfels et al. (2018)	3DCNN	Voxel size 0.94 × 0.94 × 1, FOV dimensions 196 × 256 × 160	T1-MRI	1.43 [0.03]
Hong et al. (2020)	3D CNN	“newborns (≤1 month): voxel dimensions = 1.0 × 0.7 × 4.5 mm older children (>1 month): voxel dimensions = 1.4 × 1.0 × 5.0 mm”	1.5 T T1-w MRI	67.6d, 0.971
Zhao Y. et al. (2019)	ridge regression	Not stated	1.5 T or 3 T T1-weighted	1.41 years, 0.71 1.42 years, 0.70
Liang et al. (2019)	Penalized ridge regression	Not stated	T1w MRI	6–30 years age range, 2.53 years, 0.85
	Support vector regression			Not presented
	Gaussian processes regression			Not presented
	Deep neural network			Not presented
Lewis et al. (2018)	Elastic net penalized linear regression model	resolution of 1 mm isotropic	1.5 T T1-w MRI 3 T T1-w MRI	504 d best model
Dean et al. (2015)	Voxel-wise probabilistic model	Not stated	Voxel-wise VFM maps	Males: 79.06 d Females: 90.02 d
Pardoe and Kuzniecky (2018)	Relevance vector machine regression	Not stated	T1-w MRI	7.2 y
	Gaussian processes regression			8.4 y
Lavanga et al. (2018)	Linear mixed effect regression model	n.a.	EEG	1.51 w, 0.8
Liu et al. (2024)	Graph Convolutional Network (GCN)	*UCSF:* enrolled until 2011: 1× 1 × 1 mm^3^ resolution enrolled between 2011 and 2017: 0.7 × 0.7 × 1 mm^3^ resolution	*UCSF:* Enrolled until 2011: 1.5 T T1-w MRI enrolled between 2011 and 2017: 3 T T1-w MRI	0.963 weeks, 0.94
		*dHCP:* 0.5 × 0.5 × 0.5 mm3 resolution	*dHCP:* 3 t T1-w MRI	
Tang et al. (2023)	2D Convolutional Neural Network	Not stated	T1-w MRI	1.15
	3D Convolutional Neural Network			1.80
Liu et al. (2023)	radiomics first-order grayscale feature extraction method gray matter	Not stated	1.5 T MRI	104.41
	radiomics first-order grayscale feature extraction method white matter			92.72
	FreeSurfer feature extraction method			81.83
Mendes et al. (2023)	Convolutional Neural Network	Not stated	T1-weighted MRI	0.47 [0.01], 0.18 [0.04]
Zandvoort et al. (2024)	Support Vector Regression with linear kernel function	n.a.	EEG & EMG	1.75 weeks (95% at [1.51, 2.03])
Nielsen et al. (2023)	Support Vector Regression	0.8-mm isotropic resolution	3 T T2-weighted MRI & resting-state fMRI	R2: 0.51 0.59
Hu et al. (2023)	Convolutional Neural Network on RAW data	0–6 months: slice thickness = 4.0 mm, in-plane resolution = 0.7 × 0.7 mm2 6–36 months: slice thickness = 5.0 mm, in-plane resolution = 0.7 × 0.7 mm 2	3 T T1-weighted MRI	67.66, 0.91
	Convolutional Neural Network on white matter			72.17, 0.89
Griffiths-King et al. (2023)	Gaussian Processes Regression	Not stated	T1w MRI	1.48y, 0.37
Bellantuono et al. (2021)	Deep neural network on the full dataset	Not stated	T1 weighted MRI	2.19 [0.03y], 2.91 [0.03y]
	Deep neural network on the subset of subjects within the 7–20 age range			1.53 [0.02], 1.94 [0.02]
	Deep neural network on an external dataset			2.7 [0.2], 3.7 [0.2]

y, year; m, month; w, week; MAE, Mean Absolute Error; rs-fMRI, resting-state functional MRI; RVM, Relevance vector machine; RVR, Relevance vector regression; RVM, Relevance vector machine; SVM, Support Vector Machine; SDAE, absolute error standard deviation; MSE, mean standard error.

All public datasets that were used contained MRI-based data. These are proton density, resting-state functional MRI (rs-fMRI), and structural MRI. We start with the studies that used the PING dataset for their model. Brown et al. (2012) achieved a mean prediction error of 1.03 years (age range: 3–20 years, SD: 4.9 years) using T1- and T2-weighted images in combination with diffusion MRI with a regularized multivariate nonlinear regression-like model. For resampling, they performed leave-one-out cross-validation. For comparison with the next study, the subset model containing only T1 weighted MRI images had an average prediction error of 1.71 years (age range: 3–21 years) and a correlation of 0.91. Ball et al. (2017) T1 weighted MRI in a Gaussian Process Regression model and a 10-fold cross-validation were used to determine internal model accuracy. In addition, they validated the model on the ABIDE I & II datasets. In training, they achieved 1.54 years of MAE, a correlation of 0.926 for predicted and chronological age. External model validation showed an MAE of 1.65 years and a correlation of 0.825 for ABIDE I and an MAE of 1.54 years and a correlation of 0.817 for ABIDE II. Both authors pointed out that the MAE is proportional to chronological age. Lewis et al., which relied on structural MRI, demonstrated that an elastic net penalized linear regression model yielded a MAE of 558 days (~1.53 years) (age range: 3–20 years) and 504 days (~1.38 years) (age range: 4.5–18.5 years) on the PING and NIH datasets, respectively. The model displayed optimal performance when incorporating white/grey contrast and cortical thickness for model development. The researchers noted that white/grey contrast is more crucial than thickness, but including both variables resulted in the best model accuracy (Lewis et al., 2018).

From the teams that used the National Institute of Health (NIH) Pediatric Repository (Evans, 2006), He et al. (2020) with their 2D-ResNet18 + Long short-term memory model using T1 weighted MRI images achieved the lowest average MAE with 0.96 years (age range: 0–22 years). They also applied the model to an unseen dataset, namely the Massachusetts General and Boston Children’s Hospitals (MGHBCH) dataset, which resulted in an average MAE of 1.14 (age range: 0–6 years). Additionally, they extracted the data for children aged 0–6 years. They reapplied the model, which resulted in an average MAE of 0.78 years, which strengthens the conclusion of other authors that inter-individual variance increases with age (He et al., 2020). Franke et al. used a relevance vector regression and achieved a mean absolute error of 1.1 years (age range: 5–18 years) and a correlation of 0.93 with low changes over age groups. In addition, the team applied the created model to a dataset with preterm children and was able to show negative scores, which can be interpreted as slower brain development in that cohort (Franke et al., 2012).

The following dataset that was used is the Philadelphia Neurodevelopmental Cohort (Satterthwaite et al., 2016). Erus et al. yielded the lowest MAE with 1.22 years (age range:8–22 years, SD: 3.27 years) using 3 T T1-weighted MRI images combined with diffusion tensor imaging. The model has been deployed to the PNC dataset and was validated by 10-fold cross-validation (Erus et al., 2015). Sturmfels et al. (2018) used a 3DCNN and achieved an MAE of 1.43 (age range:8–21 years). Li et al. (2018), which used a convolutional neural network with resting state functional MRI data as input, achieved the second best with an MAE of 2.15 years (age range:8–22 years) and a correlation of 0.614, but with a 5-fold cross-validation.

Eight studies used the Autism Brain Imaging Data Exchange (ABIDE) I and II. A preprocessed version of these datasets is also available at http://preprocessed-connectomes-project.org/ and has been used by Hu et al. combined with the ADHD200 dataset. The authors achieved the lowest MAE of 1.01 years using a 3DCNN for an age range of 6 to 18 years (Hu et al., 2021). Ball et al. and Pardoe et al. also used the ABIDE I&II datasets but processed the data themselves. While Pardoe and Kuzniecky (2018) only used ABIDE I&II, achieving an MAE of 7.2 years with their RVR, Ball et al. (2017) used the ABIDE datasets for validation, resulting in an MAE of 1.54 years (age range: 3-21y). Griffiths-King et al. (2023) achieved an MAE of 1.48 years (age range: 6.5–16.9) with the preprocessed ABIDE I dataset and a Gaussian Process Regression. Bellantuono et al. preprocessed the data and scored an MAE of 2.19 years for the age range of 7–64 years and 1.53 years for a subset with the age range of 7–20 years. The authors note that they perceived an improved MAE and RMSE, but Pearson’s correlation worsened. They contend that this is attributed to the heterogeneous nature of the data, stemming from acquisition at various sites within the ABIDE I dataset (Bellantuono et al., 2021). Mendes et al. (2023) used ABIDE II, ADHD-200, ABCD, and BHRCS and achieved an MAE of 1.51 for the age range of 6.1–20.0.

All studies that used EEG as input data used self-recruited patients. Vandenbosch et al. were the only authors to study children aged 5 to 18. They achieved an MAE of 1.22 years with a random forest model and 1.46 years with a relevance vector machine (Vandenbosch et al., 2019). Gschwandtner et al. showed that their model improved with higher numbers of electrodes and achieved 93.6% of estimation within ±2 weeks using 8 electrodes. The data set had an age range of 24 to 42 weeks postmenstrual age (PMA) (Gschwandtner et al., 2020). The two CT-based studies from Morita et al. were based on the same self-recruited dataset. The best model achieved an MAE of 4.61 days (age range: 0–47 months) and a correlation of 0.89 when 3D segmentation was chosen (Morita et al., 2022a).

### 3.6 Model explanation

From 51 Studies, 26 chose to inspect what parts of the input data were relevant to the final model (Ball et al., 2017, 2019, 2021; Bellantuono et al., 2021; Brown et al., 2012; Cao et al., 2015; Chen et al., 2022; Chung et al., 2018; Galdi et al., 2020; Hu et al., 2020, 2021; Tang et al., 2023; Kawahara et al., 2017; Kelly et al., 2022; Khundrakpam et al., 2015; Lewis et al., 2018; Li et al., 2018; Lund et al., 2022; Mendes et al., 2023; Nielsen et al., 2019; Qu et al., 2020; Smyser et al., 2016; Zhao T. et al., 2019; O’Toole et al., 2016; Vandenbosch et al., 2019; Lavanga et al., 2018; Morita et al., 2022a). However, the investigation of relevant parameters was performed differently, revealing a variety of approaches to this topic.

The following eight studies used the parameters themselves for importance estimation. Cao et al. segmented and then tessellated cortical and subcortical regions. Those regions were then used as input for the LASSO method. The team used leave-one-out cross-validation and created one model for each iteration, thus 303 models, and saved these sets of coefficients. The LASSO regression used these sets of coefficients and pruned the variables to regions that were used most often within the other models. This method left the new LASSO model with 37 variables that explained most of the variance. The brain stem, left thalamus, and the right lateral ventricle showed the biggest gain in volume, while the right and left Precuneus and a variable called “restralmiddlefrontal” lost most volume. Two years later, a second scan of the same cohort was scheduled, and the variables were checked for significance in a dependent t-test. It was reported that the volume in every region changed (Cao et al., 2015). Nielsen et al. used a data- and hypothesis-driven feature selection and compared these to a null model, meaning that the used variables were randomly chosen. They found that strong positive and negative-resting state functional connectivity within functional systems best predict age in their model (Nielsen et al., 2019). Chung et al. (2018) describe that their model primarily used negative weights for grey matter for increasing age. Hu et al. took a similar approach for their hierarchical-rough-to-fine model, as the importance was analyzed by relative importance, relative performance, and relative irreplaceable contribution. The relative importance was estimated by the sequential change of the exponential coefficients in the decision boundaries for the age group selection within the rough estimation of the model. To investigate the relative contribution of each feature type, they were excluded from the model, and the change was examined by mean average error, MRAE, and the 95% confidence interval (Hu et al., 2020). Li et al. (2018) used a sensitivity analysis on leave-one-out testing images for both their studies to identify important regions of interest. Khundrakpam et al. (2015) created a ranking of the best predictors of biological age based on the absolute values of the model coefficient b_i_. A similar approach was taken by Lund et al. via Correlation-Adjusted (marginal) regression (CAR) scores from the model giving a measure of variable importance (Lund et al., 2022).

Five studies used Gradient-weighted Class Activation Mapping (Selvaraju et al., 2019) (Grad-CAM), which produces a localization map presenting the important regions in the image that were used to predict specific models of the CNN family (Bellantuono et al., 2021; Hu et al., 2021; Tang et al., 2023; Qu et al., 2020; Morita et al., 2022a). Qu et al. (2020) and Hu et al. (2021) combined this approach with BrainNet to visualize and highlight the regions that their model was interested in. BrainNet is a MATLAB-based graph-theoretical network visualization toolbox that can illustrate human connectomes as ball-and-stick models (Xia et al., 2013).

Galdi et al. (2020) selected the edges assigned a non-zero coefficient in at least 99% of cross-validation folds and reported the selected connections. Kawahara et al. used a method described by Simonyan et al. (2014), in which the partial derivatives of the output of the ANN for the inspect features were computed. This is used to visualize which edges were most predictive (Kawahara et al., 2017; Simonyan et al., 2014).

Ball et al. (2017) used neighborhood preserved embedding to produce a set of basis vectors that reconstructed the original dataset, captured nonlinear relationships and provided interpretable voxel- (vertex-)wise maps of feature importance.

Shapley Additive Explanations (SHAP) were used by Ball et al. and Kelly et al. to estimate the individual-level explanations within the model. Both specified that they have used a kernel SHAP approach. Further, Ball et al. (2021) plotted the SHAP values on a semi-inflated white matter surface using ggseg3d. Kelly et al. (2022) presented the averaged mean absolute feature importance across all subjects on cortical surface representations. Chen et al. (2022) used the iNNvestigate tool (Alber, 2024; Alber et al., 2018), which uses layer-wise relevance propagation to generate an attention map.

Brown et al. used a cross-validated multivariate fitting procedure and assessed the proportion of the total explained variance of each variable for each year. They created average attention maps for each age range by averaging the maps of images within those age ranges. The combined map was created by setting each pixel with values above 0.8 to the same color (Brown et al., 2012; Alber et al., 2018). O’Toole et al. (2016) judged the predictor variables’ performance using several metrics, including bias, MSE, correlation coefficient, the standard deviation of the error in days (SD), and the standard deviation of the percentage error (SE) between the known GA and the estimated EMA. Vandenbosch et al. (2019) only performed feature importance in the random forest regression via the obtained feature importance score.

Zhao et al. attributed all voxels finding weight in the prediction models to be contributing voxels. From these, the average absolute weight in each region in the Brodmann atlas represented its importance (Zhao T. et al., 2019).

Lewis et al. used an elastic net penalized regression model and defined a signed importance. Thus, only factors had to be selected at least 50 times in the 10×10 Cross-validation. In their model, white/grey contrast and thickness were found dispersed. White/grey contrast for regions involved in low-level processing was found to be a negative factor, whereas association regions were positive. It was found to be vice versa for thickness (Lewis et al., 2018). Lavanga et al. also shrank down the model to 10 features that reached the lowest MAE when applied individually. The δ2, *θ*, *α* bands of the EEG worked best for a model used on quiet sleep state data (Lavanga et al., 2018). Mendes et al. created a gradient-based sensitivity map using an algorithm called SmoothGrad. This sensitivity map represents the features that contributed the most voxel-wise. However, the noise level and patterns are averaged before a sensitivity map is created. The impact of the perturbances in the output image produced by the input images is measured (Mendes et al., 2023; Smilkov et al., 2017).

### 3.7 Clinical application

None of the studies in this review reported using their model in a clinical routine. However, some suggested their models could be used as a clinical tool if further developed or explained the desire to implement it in a clinical setting. Tang et al. propose general usage of their model for three areas. First, as easy-to-deploy brain analysis software for clinical brain maturation assessment. Second, as a low-cost treatment tool for primary care institutions for graded care. Third, as a large-scale diagnosis tool (Tang et al., 2023). Liu et al. (2024) mention that their model used clinically relevant features, but translation is not mentioned. Franke et al. (2012) mention that future work will extend the approach, trying to identify significant regional deviations for clinical application. Gschwandtner et al. (2020) and Cao et al. (2015) also mention that clinical implications are the subject of future research. Stevenson et al. (2020) and O’Toole et al. (2016) report that the algorithm proved less error-prone than human reviewers but also state a conflict of interest. However, the remaining authors were more cautious, reporting brain age estimation as a potential biomarker, leaving pursuit unaddressed or rejecting it (Chen et al., 2022; Vandenbosch et al., 2019).

## 4 Discussion

**Figure 5:**
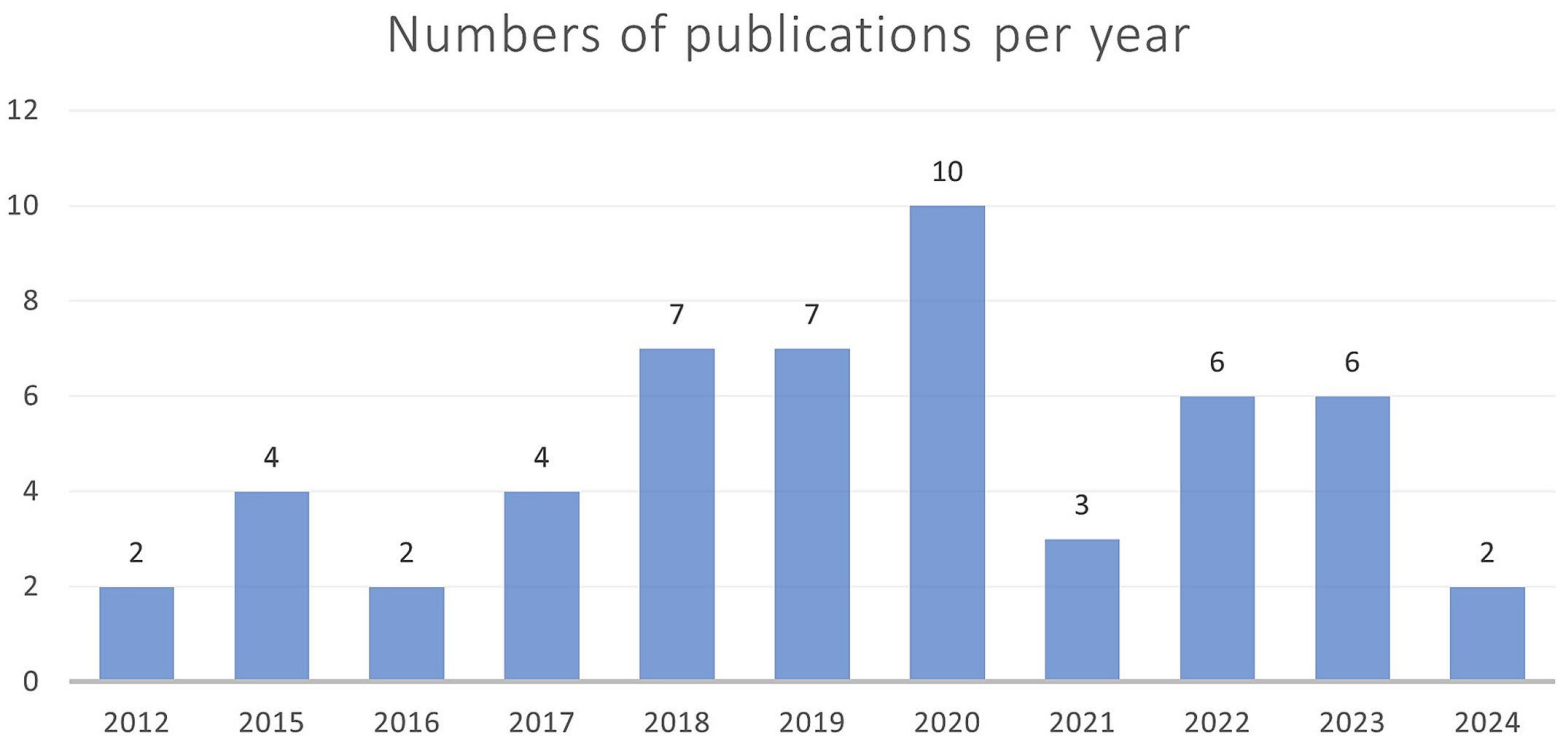
This figure shows the years in which the number of articles was published. 2020 had the highest number of publications. In 2024, only records published until April were included.

Overall, it must be noted that many studies had a specific scope that went beyond predicting the brain age with a new model. This review provides an overview of current research on using machine learning to predict children’s brain ages. It assists researchers in gaining a comprehensive understanding of past and present approaches, as well as identifying research gaps and interesting niches that are yet to be filled. Overall, the peak interested seemed to be in year 2020 as can be seen by Figure 5. It must be noted that literature of 2024 was only included until early April.

### 4.1 Data acquisition

It was found that the overall preferred type of data acquisition was MRI data. This modality is expensive and difficult to acquire compared to EEG and CT. Its acquisition is time-consuming and difficult as children are sedated or need to sleep during the procedure to prevent movement artifacts (Barkovich, 2005; Abdelhalim and Alberico, 2009; Dubois et al., 2021). Besides these disadvantages, MRI transfers most information about the maturation status of the brain. It allows for the assessment of myelination, sulcation, and chemical maturation (Barkovich, 2005). These difficulties in acquisition might explain why many studies using MRI data opted for public datasets instead of self-recruiting. As noted in the result section, 19 of 36 studies relied on publicly available data. An advantage of these datasets is that studies are comparable because they use the same data parameters if the same scan sites were chosen. Especially for fundamental research, comparability is important to prove superiority, but it also means that studies are more prone to fail in a clinical application as it introduces selection bias. This has been shown by Mendes et al. (2023), who compare the generalizability and performance of the same model architecture on distinct datasets. Comparing the demographic structure of studies using the same datasets shows that the authors chose different individuals from these datasets. Thus, even though some authors chose to use the same dataset, the data subsets, and therefore their age ranges used for training their models, still differed. Only some authors chose to validate the model on a distinct data set. As for the weighting of the MRI, most studies used T1-weighted MRI images or combined modalities, while only Smyser et al. used solely T2-weighted MRI images. This underrepresentation shows a research gap in this area. On the one hand, a valid reason to choose T1 over T2 might be that segmentation methods for the brain in T2 weighted images are less well-developed (Bae et al., 2021). However, as segmentation methods improve, this gap could be closed with future research. Generally, T1-weighted images are predominantly useful up to 6 months, but after that, T2-weighted images become more important when evaluating the myelination status until the age of 2 years (Barkovich, 2005; Girard et al., 2007; Dubois et al., 2014). Sulcation and brain volume can still be evaluated in T1, but white and grey matter contrast decreases (Barkovich, 2005). Several authors have reported MAEs falling within approximately 1–2 years of MAE. This raises concerns about the reliability of relying solely on structural T1 MRI. However, since authors like Hong et al. successfully used T1-weighted MRI to estimate brain age, with an MAE of 68 days, it can be assumed that the T1-weighted images can also be used over the six months. It is worth noting that their age range was significantly narrower (0–5 years) compared to other studies (Hong et al., 2020). It could be that the data hides patterns that are not known yet. Eleven authors combined the two MRI weightings with each other, which means that the whole spectrum of those modality-specific advantages is taken. The model has more information it can process. This approach, unfortunately, means that two sessions are needed for data collection, making data collection even more difficult and longer. For clinical application, it would be best if one weighting and one modality are sufficient for a reliable brain age prediction. According to the literature, this would leave T2-weighted images as the preferred choice for human evaluation as they might cover a wider age range (Barkovich, 2005). However, it could be that the general rules that clinicians focus on might not apply to AI-based brain age prediction. It would be interesting to see if the same models based on T1-weighted images perform the same on T2-weighted MRI for the described age ranges. It was already mentioned above that many authors opted for publicly available datasets. A drawback is that most datasets have high-resolution data, which does not reflect real-world applications. In addition, the authors did not always report the MRI’s field strength, which decreases the transparency and comprehensibility of the study. The higher the field strength of an MRI, the higher its resolution and the amount of detail it can convey. It is utterly important to report the field strength to the reader as it gives an idea of how good the input data quality was for the model. If trained in high-resolution data, models might not perform well on low-resolution MRI. Models that work better in these real-world applications might miss the chance, as they are discarded because of the better yield with high-resolution images. Initial testing on real-world data increases the chances that these models can be widely applied. All studies that used EEG data self-recruited their patients. All studies with EEG taken together had an age range of 23 to 42 weeks postmenstrual age. This might be because of the easier nature of this method. This type of data acquisition is cost-efficient and comparably easy to perform. Further, publicly available datasets could benefit model comparison in the early stages. It was found that there have been differences in the number of electrodes used for capturing brain waves, which ranged from 2 to 30 electrodes. With the higher number of electrodes, Vandenbosch et al. obtained better results but also increased the complexity of the setup. It is unclear if the increased number of electrodes, the positions of certain electrodes, or the specific setup improves the mean prediction error compared to other studies. However, all authors showed that prediction is possible, and this area might be fruitful to further follow up upon for newborns. In addition, it could be interesting to extend this research to other age ranges. Literature indicates that there are changes in EEG for young adults and adults in a small cohort study from Zappasodi et al. (2015). Ruiz de Miras et al. (2023) support this with a cohort study and were able to distinguish between schizophrenia and healthy patients. Javaid et al. (2022) also show the categorization between young and old. For implementation in a clinical setting, it is beneficial that these models can create reliable predictions with easily and cheaply obtained data to make them widely applicable. A technique used by Morita et al., computed tomography, is medically debatable. Computed tomography uses radiation to generate images. This should be used carefully, especially in children, as radiation is harmful to health (Abdelhalim and Alberico, 2009; National Research Council (US), 1990). Typically, a head CT is performed in case of trauma, and thus, traumatic brain injury ought to be diagnosed (Kambal et al., 2014). In a setting where the CT was already taken, AI might help find pathologies like intracranial bleeding or skull fracture. A CT contains less information for predicting brain maturation compared to MRI (Barkovich, 2005). Thus, exposing children to radiation for brain age estimation is rather unlikely from a future perspective because better and more harmless methods are available. However, the quick nature and high resolution are favorable characteristics of CT. A multimodal approach, meaning combining different data modalities, as Brown et al. (2012) suggested, increases the data the AI model can use. The abovementioned team argues that one modality cannot capture the developmental process (Brown et al., 2012). Combining different modalities conveys additional data, thus increasing the features the model can select from. This statement should be evaluated. The integration of low-resolution MRI and EEG represents a novel combination of modalities that has yet to be explored.

### 4.2 Age range

As for the age range, only one author, He et al., created a model covering the age range from neonates to young adults (0–22 years) and scored an astonishing MAE of 0.96 years on the test set and 1.14 years on a different, unseen dataset. Their model was a 2DCNN + lstm and showed better performance than the contested 3DResNet18. Bellantuono et al. and Liang et al. used the most comprehensive age range for their model, starting at 7 and 6 years, respectively, and including the elderly up to 64 and 89 years. Bellantuono et al. describe that the MAE increased proportionally with the age range. As they tested the model on a subset with a lower age range, the MAE dropped from 2.2 to 1.54. Liang et al. further state that younger subjects are overestimated and older are underestimated and examine this effect. According to them, the bias is not due to a particular age or age range (Liang et al., 2019). However, Bellantuono et al. also describe that the data was highly skewed to the right, thus including more patients of younger ages. The effect of shrinking the age range for better MAE must be further investigated. Most authors tried either up to the age of 3 years or from 3 years upwards. Therefore, models were limited to specific age groups, newborn to 3 or above. For clinical application, the earliest years are of particular importance. Some authors did not disclose the composition of their study population, whereas others were very descriptive, e.g., ethnicity, sex, and socioeconomic background. Zhao et al. suggest that data pre-processing should consider ethnicity because the selected atlas can affect standardized predictions, underscoring the significance of the data’s source. A standardized way of reporting should also be implemented to show that these models include various humans with different backgrounds. In an ideal situation, the AI should be trained on a dataset that reflects real-world distributions to ensure broad applicability. Further, it is vital to strengthen transparency because, ideally, each age group should be represented by the same number of individuals.

### 4.3 Pre-processing

The processing steps in the analyzed literature were similar to those of the same acquisition method. For MRI, authors followed a similar pipeline. Comparable to other research areas, all ethnicities and sexes must be represented equally. At least for gender, some studies tried to reach an equal ratio for the dataset, as can be seen in Table 2. This shows that the pipelines are already set for MRI. Morita et al. tried to leave out skull stripping for CT but failed with this approach. In MRI studies, models without skull stripping could predict age, as proven by Sturmfels et al., Lund et al., and Hong et al. Diffusion-weighted MRI also followed the same procedures, while after processing authors use different steps to combine the data with other modalities. In EEG, most authors opted for artifact removal and high- and low-pass filtering. It presents the modality with the least amount of preprocessing steps involved.

### 4.4 Artificial intelligence models

In the literature that was reviewed, two model types dominated with frequency. The most prevalent type was the kernel-based learning algorithm with a linear kernel, especially in EEG data-driven models. While most studies used a linear kernel, one radial basis and one smoothing kernel were used. The approach with a radial basis function did not present a better result than linear kernel models. However, the model with a smoothing kernel achieved a high correlation of 0.93 and a mean absolute error of 1.1 years in a dataset ranging from 5–18 years. However, it cannot be concluded that this majorly improved the model because this is not the only variable that differed. Future studies could focus on the effects of different kernels. One team, Gschwandtner et al., used the second most prominent model type, CNNs, for EEG data and scored comparably good results with a medium number of electrodes, showing that this direction might be fruitful as a future research area for EEG data-driven studies. In studies that used MRI data, 3D-CNNs were the preferred choice. This is not surprising, given the three-dimensional data from MRI or CT images.

### 4.5 Quality

The quality of the respective models is difficult to compare because few studies with the same model type used the same dataset and acquisition method. This review clustered the studies with the same dataset and compared them. Griffiths-King et al. tested their best-performing model on a different dataset and found it performing much worse. Thus, they suggest that this critical step should be integrated into future studies, and we strongly agree with this idea (Griffiths-King et al., 2023). Mendes et al. researched the generalizability of 3DCNNs on different datasets. They found that the MAE is more influenced by the center of distribution of the training set, and the correlation seems more influenced by the sample size, confounders, and the similarities between the images’ input features of the training and test set. Thus, comparable parameters are urgently needed when trained on only one dataset. However, Ball et al. (2017) used a GPR and applied an external model validation without reducing the MAE significantly. The bias due to the heterogeneity of data from multiple sites was also examined by Liang et al., which could not validate the assumption of bias. They state that the bias in their testing is more caused by regression to the mean (RTM) (Liang et al., 2019). Barnett et al. (2005) explain RTM as follows: “RTM is a statistical phenomenon that occurs when repeated measurements are made on the same subject or unit of observation. It happens because values are observed with random error.” Further, it was found that the developing brain underlies inter-individual variation that increases with age (Ball et al., 2021; Brown et al., 2012; Hu et al., 2020). Erus et al. (2015) found that biological sex is an important feature to improve prediction. However, two publications found no difference between males and females (Khundrakpam et al., 2015; Smyser et al., 2016). Sex-specific models should be evaluated in future works. We also found the combined usage of modalities. Unfortunately, no study aimed to compare directly the effects of including more than one modality, and thus, conclusions of the effect cannot be drawn as model architecture, data parameters, and age range varied throughout the literature. For EEG, Gschwandtner et al. found that the higher the number of electrodes, the better the prediction. As the prediction measurements of other studies varied, a general trend was not depictable. Zandvoort et al. combined electromyography and EEG readings but did not yield a better result than Lavanga et al., which tested two-hour EEG readings in preterm neonates. Overall, quality assessment poses a challenge due to substantial variability in data across the literature, diverse model types, and influential parameters such as field strength, resolution, and number of electrodes. Authors must persist in testing different model types and evaluating diverse datasets.

### 4.6 Model explanation

Further, ensuring the explainability of AI is of utmost importance, particularly within the medical field. We found that many AI systems operate as “black boxes,” which hinders understanding their decision-making processes. Among the included studies, only 26 out of 51 have integrated features that explain their models and choices, leaving nearly half without such provisions. Some studies have used the coefficients of variables within the models, while others have employed methods like SHAP-values, BrainNet, GradCAM, or attention maps to offer explanations. These are good examples of how it can be incorporated into modern AI. However, we also understand that articles from before 2019 could not integrate GRAD-CAM as the technique was unavailable before 2019 (Selvaraju et al., 2019). However, there has been a clear trend since then to incorporate explainability into CNNs. In 2020, only 1 out of 4 studies incorporated XAI; all studies did it in 2021; in 2022, it was 2 of 3, and in 2023, it was also 2 of 3, but the one that did not include it stated it was part of future investigations. There is an urgent need for model explanations in this research area, detached from acquisition methods. Creating a model with high precision and accuracy does not inherently render it a good and usable model, primarily when the transition to a clinical setting is pursued. For clinical decisions, it is of utmost importance that we properly understand how these models work and what they consider essential. More research must be performed for a clinical application, and more research in the areas stated above needs to be done. We must admit that the analyzed studies merely aspire to a clinical transition and are focused on the proof of principle. The field has witnessed rapid advancements in research over the past decade, with artificial intelligence (AI) demonstrating increasingly potent capabilities. Early comprehension of these models will accelerate the achievement of clinical translation. A clinical application would be cost-effective and lead to earlier detection of pathologies, which could further reduce the disease burden, as treatment could be offered earlier.

### 4.7 Verdict

All in all, further research must be done in this field. The accurate age prediction in children is still in development and it is not certain which modalities are the most promising. We think that both, EEG and MRI, have their own strengths and thus are worthwhile researching. Electroencephalography is easier to apply and cost-efficient, whereas magnetic resonance imaging conveys structural and functional information. Specific modalities, such as T2-weighted MRI, are underrepresented, which we think could be a good addition, as it conveys different information as explained in the introduction. In addition, no studies have used publicly available datasets for EEG models. Although age prediction should not be bound to specific datasets, it would enable comparison between the models. However, we want to point out that the diversity in this research field is welcomed as this research topic is quickly developing. Regarding the reporting structure for the included individuals, we recommend a standardized structure. Sex, age distribution, mean age, standard deviation, scanning sites and the corresponding scanner parameters, number of participants from these scanning sites and weightings or other settings important to the performed data generation should be structurally reported. On the one hand, the authors already clearly state the demographic structure of their data. This includes age distribution, biological sex, mean age, and standard deviation. On the other hand, some authors only state the number of participants, age range, and dataset name. This varying reporting style is inconclusive for the reader and inhibits comparability, reproducibility and transparency. The same principle applies when reporting the methods used to acquire the data. Datasets are not always clearly linked, and the scanner parameters are not always clearly stated. This is especially a hurdle when the acquisition sites differ with varying scanner parameters. Most documentation can be found online, but reporting clearly what data has been used is necessary when working with artificial intelligence and creates transparency. A clear overview of parameters for the included data would highly improve transparency. The preprocessing pipeline could be evaluated regarding the effects of skull stripping. Morita et al. indicated that the model could not be trained without brain extraction. Sturmfels et al. were able to create a model but described a decreased age prediction performance when the skull was not stripped or was too finely stripped. They suggest a regional segmentation for decreased training time and improved prediction. Further research should include this processing step as it could save resources, as suggested by Sturmfels et al. (2018). Lastly, the explainability and interpretability of the predictions will become crucial for future clinical implementation, especially in European countries where laws are getting stricter about AI. The authors of the included studies in this review partially opted for this option. The trend shows that implementation is rising among studies, and more authors describe pursuing this feature in future studies. We hope this could pave the way to a clinical application for modern medicine, thus accelerating modern and personalized medicine.

## Data Availability

The original contributions presented in the study are included in the article/supplementary material, further inquiries can be directed to the corresponding author.
